# Angiostatic, tumor inflammatory and anti-tumor effects of CXCL4^47–70^ and CXCL4L1^47–70^ in an EGF-dependent breast cancer model

**DOI:** 10.18632/oncotarget.2538

**Published:** 2014-10-21

**Authors:** Katrien Van Raemdonck, Nele Berghmans, Vincent Vanheule, Antonella Bugatti, Paul Proost, Ghislain Opdenakker, Marco Presta, Jo Van Damme, Sofie Struyf

**Affiliations:** ^1^ Laboratory of Molecular Immunology, KU Leuven - University of Leuven, Department of Microbiology and Immunology, Rega Institute for Medical Research, Leuven, Belgium; ^2^ Laboratory of Experimental Oncology and Immunology, University of Brescia, Department of Molecular and Translational Medicine Brescia, Italy; ^3^ Laboratory of Immunobiology, KU Leuven - University of Leuven, Department of Microbiology and Immunology, Rega Institute for Medical Research, Leuven, Belgium

**Keywords:** chemokines, CXCL4, CXCL4L1, EGF, CCL5, angiogenesis

## Abstract

CXCL4 and CXCL4L1, platelet-derived CXC chemokines, and their carboxy-terminal peptides CXCL4^47–70^ and CXCL4L1^47–70^ previously displayed angiostatic and anti-tumoral activity in a melanoma model. Here, we found CXCL4^47–70^ and CXCL4L1^47–70^ to inhibit lymphatic endothelial cell proliferation *in vitro*. Furthermore, the angiostatic potential of CXCL4^47–70^ and CXCL4L1^47–70^ was tested against different angiogenic stimuli (FGF1, FGF2, FGF8, EGF and VEGF). Besides reducing FGF2-induced vascular endothelial cell growth, CXCL4^47–70^ and CXCL4L1^47–70^ efficiently counteracted EGF. Consequently, we considered their anti-tumoral potential in EGF-dependent MDA-MB-231 breast tumors. In tumor-bearing mice, CXCL4^47–70^ reduced tumor growth better than CXCL4L1^47–70^. In CXCL4^47–70^-treated tumors significantly more intratumoral monocytes/macrophages and dendritic cells were present and higher expression levels of CCL5 and IFN-γ were detected by qPCR on tumor lysates. Because neither peptide was able to specifically bind CXCR3A or CXCR3B, differential glycosaminoglycan binding and direct interaction with cytokines (EGF and CCL5) might explain any differences in anti-tumoral effects. Notably, CCL5-induced monocyte chemotaxis *in vitro* was increased by addition of CXCL4^47–70^ or CXCL4L1^47–70^. Finally, CXCL4^47–70^ and CXCL4L1^47–70^ inhibited proliferation of MDA-MB-231 cells. Our results suggest a tumor type-dependent responsiveness to either CXCL4^47–70^ or CXCL4L1^47–70^ treatment, defined by anti-proliferative, angiostatic and inflammatory actions, and substantiate their therapeutic potential.

## INTRODUCTION

Angiogenesis, the formation of an ever more branching network of blood vessels from a pre-existing vascular network, has been established as a requisite for successful tumor growth and cancer progression [1, 2]. Anti-angiogenic therapy thus offers a promising approach to tackle one of the leading causes of mortality worldwide. However, classic anti-angiogenic therapeutics have encountered important hurdles and interest in less obvious targets, including inflammatory mediators such as the chemokines, increases [3–7]. These chemotactic cytokines play an important role in immunity as they control activation and migration of particular subsets of leukocytes. Chemokines have been implicated though in a variety of other biological processes, including angiogenesis [8, 9]. A distinction should be made between angiogenic (mostly CC and ELR-positive CXC chemokines) and angiostatic chemokines (mostly ELR-negative CXC chemokines). The latter group comprises the CXCR3 ligands, namely CXCL9/Monokine induced by interferon-γ (Mig), CXCL10/Interferon-γ-induced protein 10 (IP-10), CXCL11/Interferon-inducible T-cell α chemoattractant (I-TAC), CXCL4/Platelet factor-4 (PF-4) and CXCL4L1/PF-4variant. These CXCR3 ligands inhibit endothelial cell proliferation and migration, favoring the delicate angiogenic balance to shift towards angiostasis [10, 11]. In keeping with a long line of research within our group, the focus of this study lies with the platelet products CXCL4 and CXCL4L1. These non-allelic variants are both secreted by activated platelets as 70 amino acid-long mature proteins, differing only in 3 residues situated near the carboxy-terminal end (Pro^58^→ Leu^58^, Lys^66^→ Glu^66^, Leu^67^→ His^67^ for CXCL4→ CXCL4L1) [12]. Despite the limited amino acid substitutions, the extent to which these affect protein structure, and subsequently biological function, is remarkable. As a single replacement, the shift from leucine on position 67 in CXCL4 to histidine in CXCL4L1 causes the carboxy-terminal helix to protrude into the aqueous space thereby exposing the entire helix [13]. This unique carboxy-terminal 3D-structure of CXCL4L1 correlates with a decrease in glycosaminoglycan (GAG) affinity and a more outspoken angiostatic potential [13]. CXCL4L1 was demonstrated to inhibit both CXCL8- and basic fibroblast growth factor (FGF2)-induced endothelial cell chemotaxis more efficiently than CXCL4. However, CXCL4L1 is less likely to form heterodimers or compete for GAG binding [14–16]. Interestingly, carboxy-terminal peptides of both CXCL4 and CXCL4L1 have been illustrated to preserve the angiostatic and anti-tumoral potential [17–19]. CXCL4- and CXCL4L1-derived peptides are excellent probes to unravel the working mechanism of these angiostatic chemokines. A better understanding could benefit future therapeutic use of CXCL4, CXCL4L1 or their derived peptides. Here, we further explored the qualities of the carboxy-terminal peptides CXCL4^47–70^ and CXCL4L1^47–70^ as anti-tumoral agents, more specifically against a human epidermal growth factor (EGF)-dependent tumor cell line, MDA-MB-231. Our *in vitro* data support the hypothesis that both CXCL4^47–70^ and CXCL4L1^47–70^ retain their angiostatic potential. Both peptides were shown to inhibit proliferation of microvascular endothelial cells, including lymphatic endothelial cells. We also demonstrated MDA-MB-231 tumor cells to be sensitive to the anti-proliferative effect of CXCL4^47–70^ and CXCL4L1^47–70^ *in vitro*. Surprisingly, mostly CXCL4^47–70^ exerted an anti-tumoral effect on EGF-dependent MDA-MB-231 tumor growth *in vivo*.

## RESULTS

### Anti-proliferative effect of CXCL4^47–70^ and CXCL4L1^47–70^ on mitogen-stimulated bovine endothelial cells

**Figure 1:**
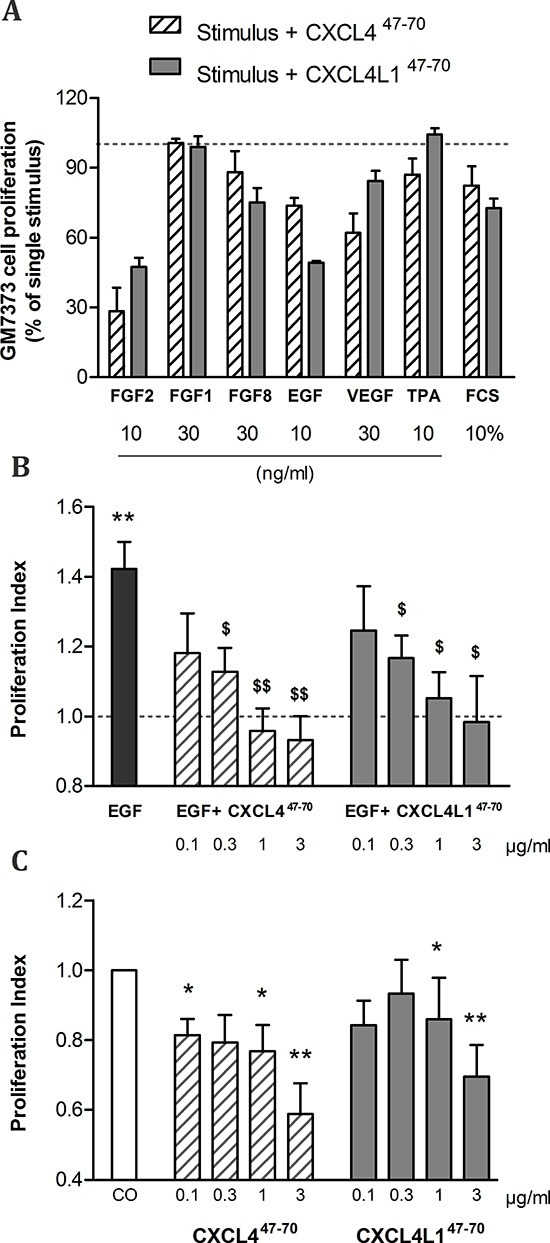
Effects of CXCL4^47–70^ and CXCL4L1^47–70^ on endothelial cell proliferation Firstly, bovine aortic endothelial GM7373 cells were incubated with a range of mitogenic stimuli either in the presence or absence of CXCL4^47–70^ or CXCL4L1^47–70^ (both 0.4 μg/ml) for 24 h **(A)**. Cell counts after incubation with one of the carboxy-terminal peptides were expressed as percentages (mean ± s.e.m.), relative to cell counts after stimulation with the indicated mitogenic stimulus alone (100%; dotted line). As only 3 independent experiments were included in this preliminary screening, no statistical significance was reached. HMVEC were stimulated with either control medium (CO), CXCL4^47–70^ or CXCL4L1^47–70^ (both 0.1 to 3 μg/ml), combined with 3 ng/ml EGF **(B)** or as single stimuli **(C)** over the course of 3 to 4 days. Afterwards plates were developed according to MTT assay protocol. Optical density was determined at 570 nm and 630 nm. The proliferation index in condition X (mean ± s.e.m.) represents the ratio of the calculated ΔOD570–630_X_ over ΔOD570–630_CO_, in which control treatment serves as an internal reference (PI= 1; dotted line). A: n= 3; B and C: n= 6 to 7; *p<0.05, **p<0.01 (*versus* CO); $p<0.05, $$p<0.01 (*versus* EGF)

Vandercappellen *et al.* demonstrated the anti-tumoral effects of carboxy-terminal peptides derived from CXCL4 and CXCL4L1 and illustrated that both CXCL4^47–70^ and CXCL4L1^47–70^ retained the ability to block FGF2-induced endothelial cell motility and proliferation [19]. We investigated whether CXCL4^47–70^ and CXCL4L1^47–70^ counteracted other growth factors with similar efficiency. A screening was performed on bovine aortic endothelial cells (GM7373) as shown in Figure 1A. Consistent with previous work [19], both CXCL4^47–70^ and CXCL4L1^47–70^ (0.4 μg/ml) reduced FGF2-induced GM7373 proliferation to 28 ± 10% and 47 ± 4%, respectively. The peptides' effect on epidermal growth factor (EGF) stimulation also stood out as CXCL4^47–70^ reduced GM7373 proliferation to 74 ± 3%. Remarkably, CXCL4L1^47–70^ consistently inhibited EGF's mitogenic activity more efficiently, lowering EGF-stimulated proliferation to 49 ± 1%.

### CXCL4^47–70^ and CXCL4L1^47–70^ inhibit human endothelial cell proliferation

Our preliminary proliferation screenings suggested angiogenic EGF stimulation to be particularly sensitive to addition of CXCL4L1^47–70^. Interestingly, EGF does not rely on glycosaminoglycans (GAG) as co-receptors to exert its activity. Furthermore, though intact CXCL4 has been reported to counteract this growth factor, its mode of action has yet to be fully unraveled [20]. We examined proliferation of human retinal microvascular endothelial cells (HMVEC) when stimulated with EGF as opposed to a combination of EGF and CXCL4^47–70^ or CXCL4L1^47–70^ (Figure 1B). As expected, EGF (3 ng/ml) stimulated HMVEC proliferation in an MTT assay with a proliferation index (PI) of 1.42 ± 0.08 (n= 7). Further addition of CXCL4^47–70^ caused the PI to drop dose-dependently (PI= 1.13 ± 0.07, p= 0.015, n= 7; PI= 0.96 ± 0.06, p= 0.003, n= 7; PI= 0.93 ± 0.07, p= 0.008, n= 6 at 0.3, 1 and 3 μg/ml, respectively). Similarly, CXCL4L1^47–70^ also significantly reduced EGF-induced proliferation (PI= 1.17 ± 0.06, p= 0.041; PI= 1.05 ± 0.07, p= 0.015; PI= 0.98 ± 0.13, p= 0.041 at 0.3, 1 and 3 μg/ml, respectively; n= 7).

As single stimulus (without growth factor stimulation) CXCL4^47–70^ and CXCL4L1^47–70^ significantly reduced baseline proliferation (Figure 1C). CXCL4^47–70^ reduced proliferation to a PI of 0.77 ± 0.08 (p= 0.011, n= 8) and 0.59 ± 0.09 (p= 0.001, n= 6) at 1 μg/ml and 3 μg/ml, respectively. Similarly, the PI was lowered to 0.86 ± 0.12 (p= 0.043, n= 8) and 0.70 ± 0.09 (p= 0.009, n= 7) after stimulation with the variant CXCL4L1^47–70^ at 1 μg/ml and 3 μg/ml, respectively. The effect of CXCL4^47–70^ on constitutive HMVEC proliferation was more prominent than that of CXCL4L1^47–70^.

### CXCL4^47–70^ and CXCL4L1^47–70^ induce cell cycle arrest in HMVEC

**Figure 2:**
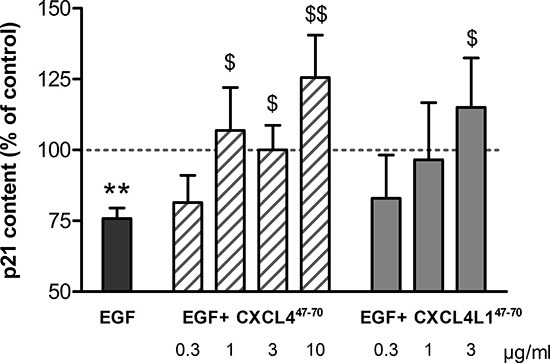
p21 content in HMVEC after CXCL4^47–70^ and CXCL4L1^47–70^ stimulation The impact of CXCL4^47–70^ and CXCL4L1^47–70^ on the cell cycle in HMVEC was evaluated by quantification of p21 production. Intracellular p21 concentrations, known to drop upon EGF stimulation (20 ng/ml), were also evaluated in HMVEC stimulated with EGF combined with either CXCL4^47–70^ (0.3 to 10 μg/ml) or CXCL4L1^47–70^ (0.3 to 3 μg/ml). The ratio of p21: total protein content was calculated for cell lysates after 15h incubation with various stimuli. Results (mean ± s.e.m.) are expressed relative to the p21 content after control treatment (100%; dotted line). n= 4 to 9; **p<0.01 (EGF *versus* CO); $p<0.05,$$p<0.01 (*versus* EGF)

Previously, the EGF-induced reduction of the cyclin-dependent kinase inhibitor p21 was described [20]. Interestingly, CXCL4 appeared to interfere with this p21 downregulation in EGF-stimulated endothelial cells. Therefore, we studied the effects of chemokine-derived peptides on p21 levels and, with it, their ability to arrest the cell cycle. Evaluation of the p21 content in HMVEC did confirm CXCL4^47–70^ and CXCL4L1^47–70^ to counteract the tendency of EGF to reduce p21 (Figure 2). EGF (20 ng/ml) reduced intracellular p21 to 75.82 ± 3.69% (n= 9; p< 0.001) compared to control-treated levels. Addition of the peptides in combination with EGF restored p21 levels to control levels (i.e. p21 levels in the cells treated with peptide plus EGF were not statistically different from buffer-treated cells). After adding 1, 3 or 10 μg/ml CXCL4^47–70^, p21 levels were also significantly higher than those in HMVEC stimulated solely with 20 ng/ml EGF (106.90 ± 15.12%, p= 0.039, n= 6; 100.02 ± 8.67%, p= 0.039, n= 9; and 125.51 ± 15.05%, p= 0.005, n= 5, respectively). Stimulation with 3 μg/ml CXCL4L1^47–70^ significantly restored EGF-reduced p21 levels to 115.06 ± 17.41% (p= 0.033, n= 5).

### CXCL4^47–70^ and CXCL4L1^47–70^ reduce lymphatic endothelial cell proliferation

**Figure 3:**
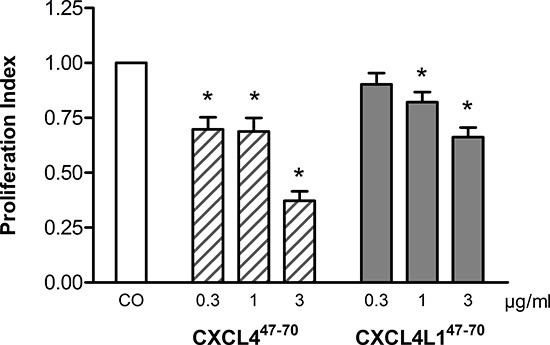
Effect of CXCL4^47–70^ and CXCL4L1^47–70^ on proliferation of lymphatic endothelial cells In order to study proliferation of human lymphatic endothelial cells, HLEC were stimulated with either control medium treatment (CO), CXCL4^47–70^ or CXCL4L1^47–70^ (both 0.3 to 3 μg/ml). After an incubation period of 3 to 4 days, cell cultures were analyzed with the MTT assay protocol. n= 4; *p<0.05 (*versus* CO)

Only recently, the link between the angiostatic CXCR3 ligands and lymphangiogenesis was recognized [21–23]. As the lymphatic vasculature has been implied to be an escape route for metastatic tumor cells, its blockade offers an interesting new therapeutic perspective [24]. Though proliferation of human dermal lymphatic microvascular endothelial cells (HLEC) was not responsive to EGF-stimulation (data not shown), baseline proliferation was indeed sensitive to CXCL4^47–70^ and CXCL4L1^47–70^ (Figure 3). CXCL4^47–70^ reduced the PI to 0.70 ± 0.06 (p= 0.030), 0.69 ± 0.06 (p= 0.030) and 0.37 ± 0.04 (p= 0.030) at 0.3, 1 and 3 μg/ml, respectively (n= 4). The variant peptide CXCL4L1^47–70^ reduced the PI less efficiently to 0.90 ± 0.05 (p= 0.312, n= 4), 0.82 ± 0.05 and 0.66 ± 0.04 (both p= 0.030, n= 4) at 0.3, 1 and 3 μg/ml, respectively.

### CXCL4^47–70^ and CXCL4L1^47–70^ reduce proliferation of MDA-MB-231 cells

**Figure 4:**
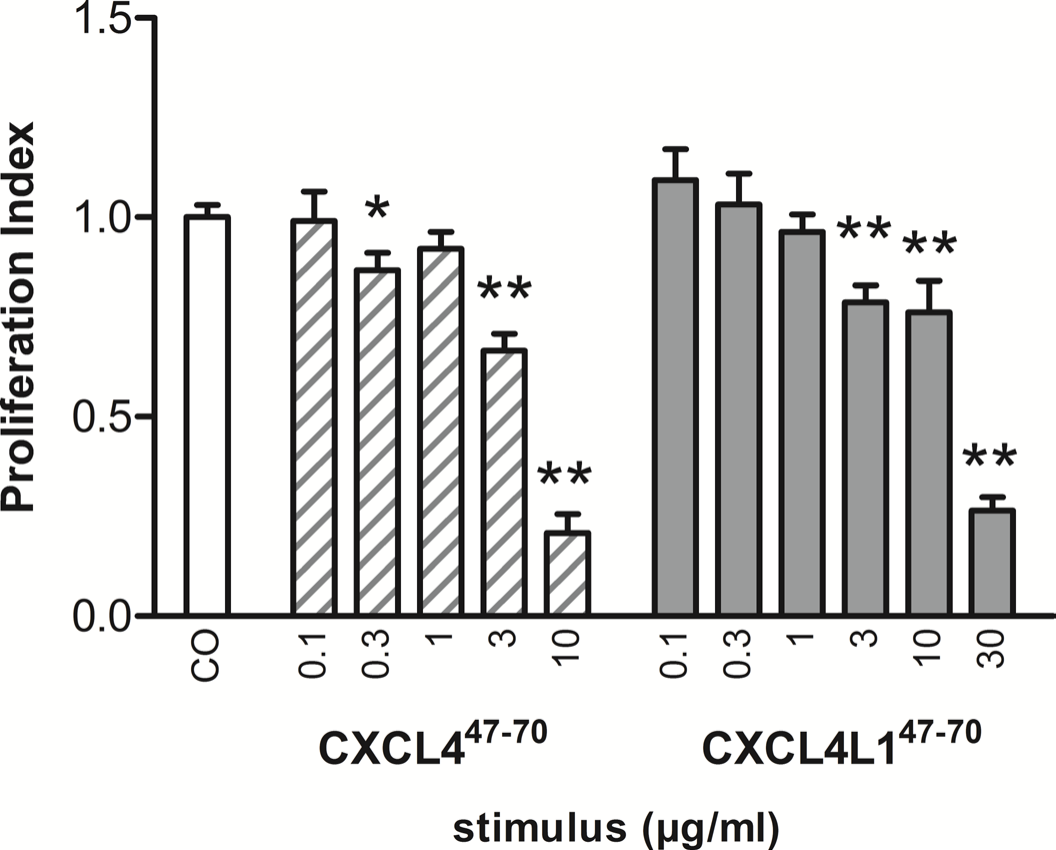
Proliferation of MDA-MB-231 cells examined after CXCL4^47–70^ and CXCL4L1^47–70^ stimulation The anti-proliferative effect of CXCL4^47–70^ and CXCL4L1^47–70^ on MDA-MB-231 tumor cells *in vitro* was investigated performing the MTT proliferation assay. Over the course of 3 to 4 days, MDA-MB-231 cells were incubated with either control treatment (CO), CXCL4^47–70^ (0.1 to 10 μg/ml) or CXCL4L1^47–70^ (0.1 to 30 μg/ml). Afterwards cell cultures were analyzed with the MTT assay protocol. n= 3 to 7, tested in triplicate per experiment; *p<0.05, **p<0.01 (*versus* CO)

While most of the anti-tumoral effects of CXCL4 and CXCL4L1 have been attributed to their angiostatic potential, little attention has been paid to the direct anti-proliferative effect they may exert on malignant cells themselves [25]. We evaluated proliferation of MDA-MB-231, EGF-dependent breast cancer cells lacking estrogen and progesterone receptor expression as well as human EGF-receptor 2 (HER2) amplification (triple-negative) [26, 27]. Our results show that CXCL4^47–70^ and CXCL4L1^47–70^ were able to significantly and dose-dependently reduce MDA-MB-231 proliferation (Figure 4). The PI was lowered to 0.87 ± 0.04 (p= 0.023, n= 7), 0.66 ± 0.04 (p< 0.001, n= 7) and 0.21 ± 0.05 (p< 0.001, n= 3) at 0.3, 3 and 10 μg/ml of CXCL4^47–70^, respectively (all conditions tested in triplicate). This indicates that high doses of CXCL4^47–70^ have a potent direct anti-proliferative effect on MDA-MB-231 cells. Increasing doses of CXCL4L1^47–70^ also reduced the PI to 0.78 ± 0.04, 0.76 ± 0.08 and 0.26 ± 0.04 (p< 0.001, n= 7 at 3 μg/ml; p= 0.01, n= 7 at 10 μg/ml; and p< 0.001, n= 3 at 30 μg/ml, respectively; tested in triplicate). We attempted to corroborate these data by assessment of the intracellular p21 content, yet found this p53-dependent signaling protein to fall below the detection limit of the available assays (data not shown) [28].

### CXCL4^47–70^ and CXCL4L1^47–70^ reduce MDA-MB-231 tumor growth *in vivo*

**Figure 5:**
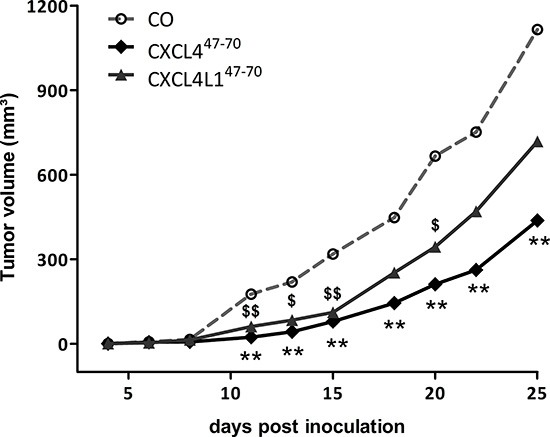
Tumor growth in CXCL4^47–70^- and CXCL4L1^47–70^-treated tumor-bearing mice Progressive tumor growth was assessed in SCID mice, subcutaneously inoculated with MDA-MB-231 tumor cells on day 0 and subsequently treated intratumorally with vehicle (CO), CXCL4^47–70^ or CXCL4L1^47–70^ (2.5 μg; 3×/week from day 3 onwards). The tumor volume was measured externally with calipers thrice a week and calculated using the formula (4πab2)/3 in which a and b are the largest and smallest radius, respectively. Growth curves represent increasing mean tumor volumes. n=10/group; **p<0.01 (CXCL4^47–70^ *versus* CO); $p<0.05, $$p<0.01 (CXCL4L1^47–70^ *versus* CO)

In order to evaluate the sum of their angiostatic and anti-tumoral effects, we assessed the *in vivo* relevance of intratumorally administered CXCL4^47–70^ and CXCL4L1^47–70^. Following up on s.c. MDA-MB-231 tumor growth through external examination (Figure 5), we noted a distinct reduction in tumor volume in CXCL4^47–70^-treated versus vehicle-treated mice from 11 days after tumor cell inoculation onward (24 ± 6 mm^3^ and 176 ± 56 mm^3^, respectively; p< 0.001, both n=10). At early stages up till around 20 days after tumor cell inoculation, the CXCL4L1^47–70^-treated mice also displayed significantly smaller tumors than their vehicle-treated counterparts (61 ± 23 mm^3^ on day 11, p= 0.007, n=10). However, after 25 days CXCL4L1^47–70^ appeared less effective than CXCL4^47–70^ in the treatment of MDA-MB-231 tumors. At day 25, the mean tumor volume in the vehicle-treated mice amounted to 1116 ± 149 mm^3^, whereas CXCL4^47–70^ treatment had efficiently reduced tumor growth with a mean tumor volume of 438 ± 97 mm^3^ (p= 0.002, both groups n= 10). The effect of CXCL4L1^47–70^ was not statistically different from the control group (719 ± 153 mm^3^, p= 0.121, n= 10).

**Figure 6:**
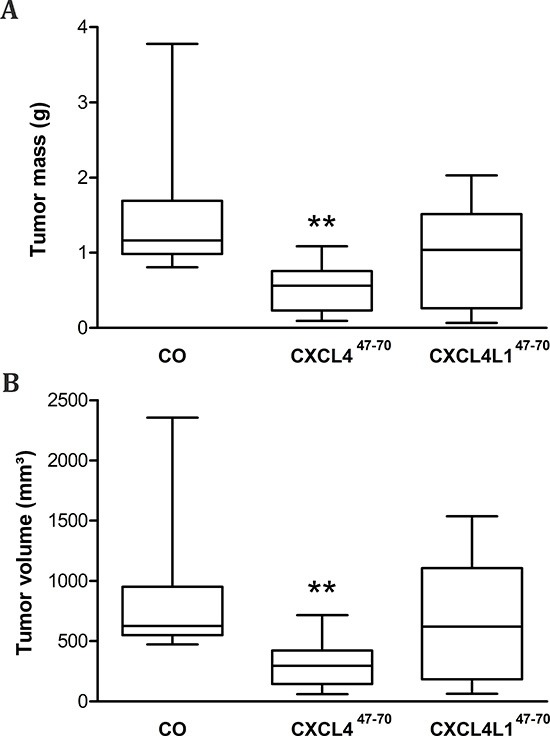
Tumor volume and mass after periodical CXCL4^47–70^ and CXCL4L1^47–70^ treatment After vehicle (CO), CXCL4^47–70^ or CXCL4L1^47–70^ treatment (2.5 μg; 3×/week) of MDA-MB-231 tumor-bearing mice, on day 27 or 28 resected tumors were weighed **(A)** and tumor volumes **(B)** were determined (4πabc/3 in which a,b and c represent measured radii). Data were depicted as a 5–95 percentile Whiskers plot, in which the horizontal line marks the median value. n=10/group; **p<0.01

Afterwards, 27 to 28 days after initial MDA-MB-231 cell injection, masses and volumes of resected tumors were compared among the different treatment groups (Figure 6). Average tumor volume among vehicle-treated animals amounted to 845 ± 177 mm^3^, whereas after periodical intratumoral injection of CXCL4^47–70^ average tumor volume was reduced to 308 ± 61 mm^3^ (p= 0.001, n= 10). Total tumor mass was also reduced from 1464 ± 277 to 523 ± 108 mg on average (p= 0.002, n= 10). Administration of CXCL4L1^47–70^ did not significantly alter tumor volume, nor mass (647 ± 161 mm^3^, p= 0.256 and 953 ± 208 mg, p= 0.091, respectively; both n= 10) after 4 weeks.

### Vascularization of CXCL4^47–70^- and CXCL4L1^47–70^- treated tumors

The impact of peptide treatment on the tumor vasculature was evaluated by quantification of immunohistochemically stained CD31-positive vessels. However the angiostatic activity of neither CXCL4^47–70^, nor CXCL4L1^47–70^ was reflected by a reduced vascularization of the resected MDA-MB-231 tumors (data not shown). This inconsistency between our observations *in vitro* and *in vivo* evokes the question which alternative mechanisms may contribute to the anti-tumoral impact of peptide treatment as evidenced in the MDA-MB-231 tumor model.

### Tumor inflammation in CXCL4^47–70^- and CXCL4L1^47–70^-treated MDA-MB-231 tumors

**Figure 7:**
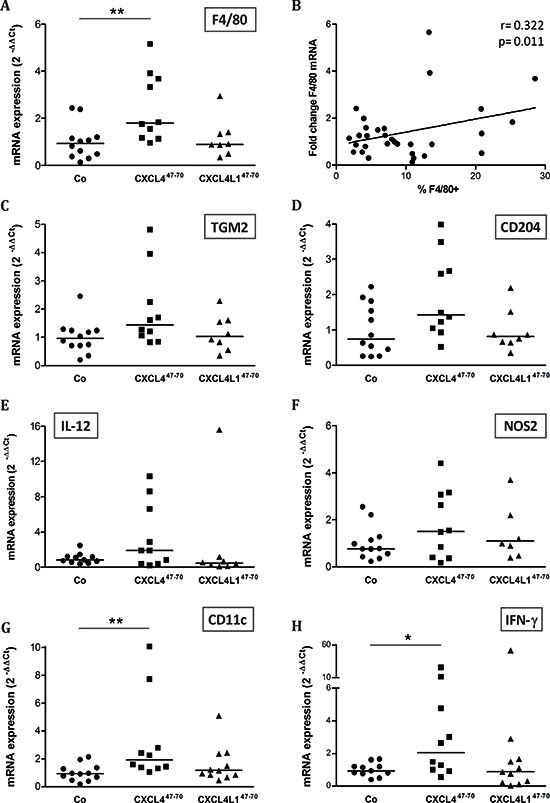
Effects of intratumoral CXCL4^47–70^ and CXCL4L1^47–70^ administration on the intratumoral inflammatory infiltrate Relative expression of macrophage marker F4/80 was evaluated in vehicle (CO)-, CXCL4^47–70^- and CXCL4L1^47–70^-treated MDA-MB-231 tumors by qPCR **(A)**. Additionally, the relative F4/80 mRNA levels were plotted in function of the percentage of F4/80^+^ cells as determined by flow cytometry **(B)**. Panel B includes the calculated Pearson's correlation coefficient (r) and according p-value. In parallel, changes in the expression of M2 markers TGM2 and CD204 **(C and D**, respectively) and M1 markers IL-12 and NOS2 **(E and F**, respectively) were also analyzed by qPCR. Finally, expression of inflammatory markers CD11c **(G)** and IFN-γ **(H)** were compared between groups. Fold changes in mRNA levels were calculated according the 2^−ΔΔCT^ method. The median value per group is indicated by a horizontal bar. *p<0.05, **p<0.01

Although the used SCID mice are characterized by defective T-lymphocyte development, monocyte, macrophage and NK cell populations have been shown to be unaffected in this mouse strain. We investigated possible changes in the immunological tumor microenvironment as a consequence of CXCL4^47–70^ or CXCL4L1^47–70^ treatment. Expression levels of various macrophage markers [29–31] were evaluated, all showing on average an elevated mRNA expression in the CXCL4^47–70^-treated group (Figure 7). However, only F4/80 mRNA levels were significantly higher within resected tumors of CXCL4^47–70^- treated mice compared to control mice (p= 0.008, n= 10; Figure 7A). These data indicate that intratumorally administered CXCL4^47–70^ may favor local monocyte accumulation, as also evidenced by flow cytometric analysis (Co: 6.85 ± 1.18% F4/80^+^ cells, n= 10; CXCL4^47–70^: 7.70 ± 1.58% F4/80^+^ cells, n= 9). Overall, enhanced F4/80 mRNA levels indeed statistically correlated to an increased number of intratumoral F4/80^+^ cells (r=0.322 and p=0.011) (Figure 7B). Additionally, specific markers for M1 and M2 macrophage polarization were evaluated. TGM2 (Figure 7C) and CD204 (Figure 7D) are both specific markers of the pro-tumoral M2 phenotype, as opposed to F4/80 which is a general macrophage marker. These M2 markers exhibited only a modest increase after CXCL4^47–70^ treatment. M1 markers IL-12 (Figure 7E) and NOS2 (Figure 7F) also only modestly increased after CXCL4^47–70^ treatment, not reaching statistical significance. Nevertheless, M1-related IL-12 upregulation seems more pronounced than that of TGM2 or CD204. Collectively, these observations suggest that the number of both M1 and M2 macrophages increases and that there is no dominance of a specific subtype. No significant changes in macrophage marker expression were observed in CXCL4L1^47–70^- versus vehicle-treated mice. Flow cytometric analysis of the tumor stroma did, however, suggest that CXCL4L1^47–70^ also recruited macrophages (CXCL4L1^47–70^: 7.74 ± 1.35% F4/80^+^ cells, n= 9). In order to corroborate and further explore the inflammatory effect of our peptides, tumoral expression of the dendritic cell marker CD11c was evaluated (Figure 7G). CD11c mRNA was also more prominent in CXCL4^47–70^-treated compared to vehicle-treated mice (p= 0.032, n= 10). We additionally evaluated intratumoral expression of interferon-γ (IFN-γ). This cytokine could promote endogenous expression of the CXCR3 ligands CXCL9, CXCL10 and CXCL11, which in turn are anti-tumoral, angiostatic chemokines [11]. As shown in Figure 7H, IFN-γ mRNA was most prominent following CXCL4^47–70^ treatment. The significantly elevated expression of the inflammatory cytokine IFN-γ possibly promotes anti-tumoral immunity (cytotoxicity, phagocytosis) thereby contributing to the anti-tumoral effect of CXCL4^47–70^ *in vivo*.

### Cellular receptors of CXCL4^47–70^ and CXCL4L1^47–70^

We further tried to reveal the mode of action of CXCL4^47–70^ and CXCL4L1^47–70^. First, we excluded that either peptide still binds/activates one of the CXCR3 isoforms, the receptors for intact CXCL4 and CXCL4L1. We performed binding assays using 3 to 3000 ng/ml of NH_2_-terminally biotinylated peptides and could not detect specific binding to transfected CHO/CXCR3A, nor CHO/CXCR3B cells by flow cytometric analysis (data not shown). Nor could we detect any calcium signal in CXCR3A-transfectants after treatment with 2 μg/ml of CXCL4^47–70^ or CXCL4L1^47–70^ (Supplemental Figure 1). Furthermore, neither peptide (2 μg/ml) was capable of inhibiting calcium signaling by intact CXCL10 (5 ng/ml), added to the cells as a second stimulus 100 s after addition of one of the peptides.

**Figure 8:**
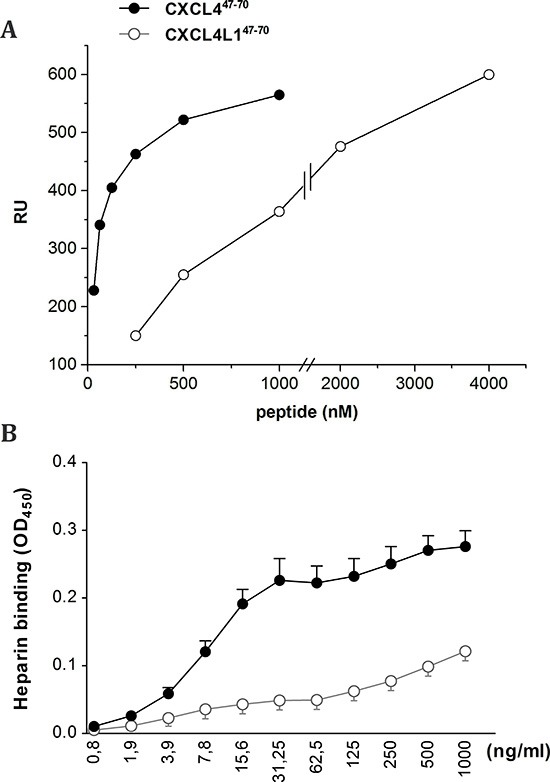
Interaction of CXCL4^47–70^ and CXCL4L1^47–70^ peptides with immobilized heparin Binding of the indicated concentrations of CXCL4^47–70^ and CXCL4L1^47–70^ to heparin was analyzed through measurement of peptide binding to sensorchip-immobilized heparin **(A)**. Data are expressed as Resonance Units (RU). Alternatively, GAG binding was evaluated by immobilizing low molecular weight heparin on EpranEx plates **(B)**. Amino-terminally biotinylated CXCL4^47–70^ and CXCL4L1^47–70^ interacting with the heparin-coated plate were detected with peroxidase-conjugated streptavidin. Results are expressed as average optical densities (± s.e.m.) measured at 450 nm (OD_450_). A: one representative experiment out of three; B: n= 3

Next, we exploited surface plasmon resonance (SPR) analysis to assess the capacity of CXCL4^47–70^ and CXCL4L1^47–70^ to bind heparin immobilized to a BIAcore sensorchip, a model that mimics the binding of proteins to heparan sulphate proteoglycans associated to the cell surface [32]. When increasing concentrations of the two peptides were injected onto the heparin surface, sensorgram overlays (not shown) allowed the calculation of association (*K_on_*) and dissociation (*K_off_*) rates and of *K_d_* (as *K_off_*/K*_on_* ratio, Table 1). Also, equilibrium binding data were used to generate the saturation curves shown in Figure 8A and used to calculate a *K_d_* value independently from kinetic parameters (Table 1). Together, the data indicate that CXCL4^47–70^ binds to immobilized heparin with high affinity (*K_d_*= 16–72 nM) whereas CXCL4L1^47–70^ showed a very limited ability to bind the immobilized glycosaminoglycan (*K_d_*= 1000–1200 nM).

**Table 1:** Binding parameters of the interaction of CXCL4^47–70^ and CXCL4L1^47–70^ peptides with heparin immobilized on a BIAcore sensorchip

Peptide	Association rate (*K_on_*) (1/Ms)	Dissociation rate (*K_off_*) (1/s)	*K_d_* (*K_off_/K_on_*) (*M*)	*K_d_* (M) at equilibrium
CXCL447–70	6.7 × 10^5^	1.1 × 10^−^2	1.6 × 10^−^8	7.2 × 10^−^8
CXCL4L147–70	5.2 × 10^4^	5.3 × 10^−^2	1.0 × 10^−^6	1.2 × 10^−^6

*K_on_* and *K_off_* of peptide/heparin interactions are shown. The *K_d_* value was derived from the *K_off_/K_on_* ratio as well as by Scatchard plot analysis of the equilibrium binding data.

We could confirm the results from the SPR analysis in an alternative GAG binding assay performed in microtiter plates coated with heparin (Figure 8B). Dilution series of biotinylated CXCL4^47–70^ and CXCL4L1^47–70^ were added to investigate heparin affinity. Also in this assay less biotinylated CXCL4L1^47–70^ peptide bound to the heparin-coated plate compared to biotinylated CXCL4^47–70^, indicative of either a lower affinity of CXCL4L1^47–70^ for heparin or a different stoichiometry of interaction.

### Interaction of CXCL4^47–70^ and CXCL4L1^47–70^ with EGF and CCL5

**Figure 9:**
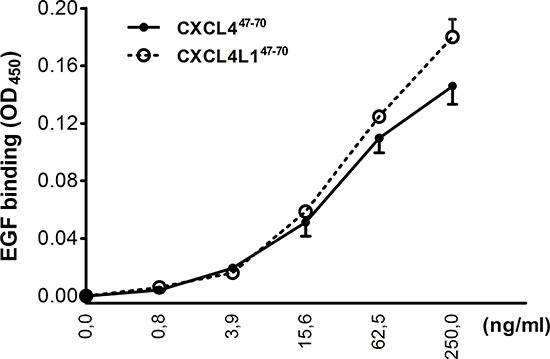
Interaction of CXCL4^47–70^ and CXCL4L1^47–70^ with EGF In order to evaluate interaction of CXCL4^47–70^ and CXCL4L1^47–70^ with EGF, binding of amino-terminally biotinylated CXCL4^47–70^ or CXCL4L1^47–70^ to EGF-coated plates was detected with peroxidase-conjugated streptavidin. Results are expressed as average optical densities (± s.e.m.) measured at 450 nm (OD_450_). n= 3

Following studies by Nesmelova *et al.* [33] and von Hundelshausen *et al.* [34], we explored whether CXCL4^47–70^ or CXCL4L1^47–70^ could also directly interact with other cytokines. In view of the role of EGF in the MDA-MB-231 breast tumor model, we first evaluated the ability of either peptide to bind to this growth factor (Figure 9). EGF heterodimerization assays revealed EGF to retain both CXCL4^47–70^ and CXCL4L1^47–70^ equally well in a dose-dependent manner. These findings may present a plausible explanation for the direct anti-proliferative effect of both peptides on the EGF-dependent MDA-MB-231 tumor cells *in vitro*.

**Figure 10:**
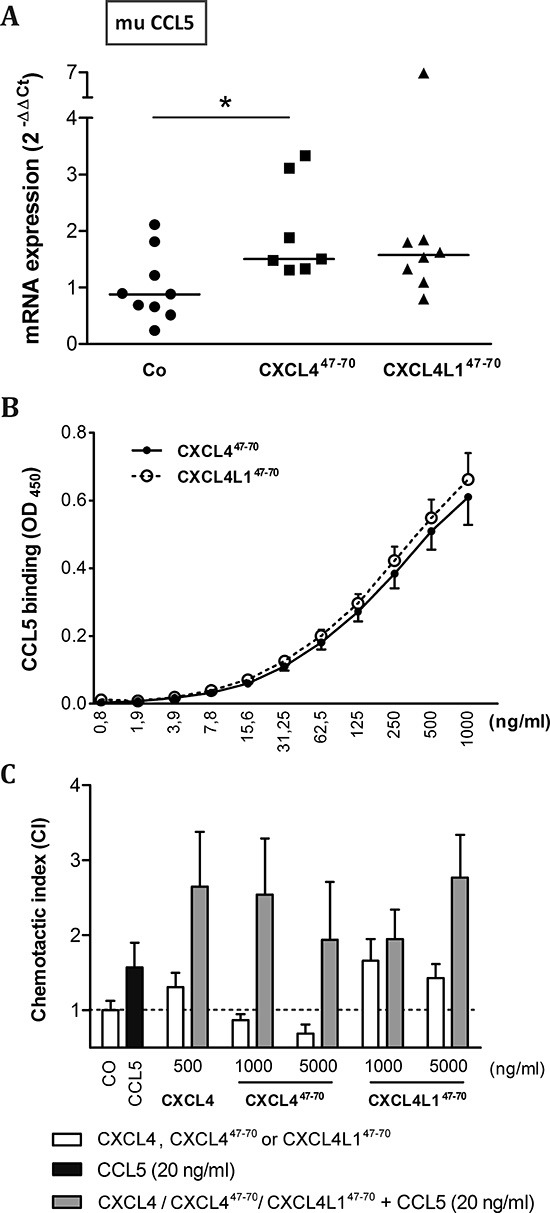
Interaction of CXCL4^47–70^ and CXCL4L1^47–70^ with CCL5 Changes in the expression of murine (mu) CCL5 within the tumor microenvironment were evaluated in vehicle (CO)-, CXCL4^47–70^- and CXCL4L1^47–70^-treated MDA-MB-231 tumors by qPCR **(A)**. Fold changes in mRNA levels were calculated according the 2^−ΔΔCT^ method. The median value per group is indicated by a horizontal bar. In order to further evaluate a possible interaction between CCL5 and the administered peptides, binding of biotinylated CXCL4^47–70^ or CXCL4L1^47–70^ to human CCL5-coated plates was quantified **(B)**, whereas functional synergy was investigated by evaluating chemotaxis of monocytic cells **(C)**. THP-1 cells were stimulated with CCL5 (20 ng/ml), intact CXCL4 (500 ng/ml), CXCL4^47–70^, CXCL4L1^47–70^ (both 1000 to 5000 ng/ml) or combinations thereof in a multiscreen chemotaxis assay. A: n= 7 to 9; B: n= 3; C: n= 3 to 4; *p<0.05

On the other hand, qPCR analysis showed the MDA-MB-231 tumor stroma to express muCCL5 (Figure 10A), a monocyte chemotactic chemokine. Stromal CCL5 expression was significantly upregulated in CXCL4^47–70^-treated mice (p= 0.020, n= 7). MDA-MB-231 cells themselves were also observed to express CCL5 *in vitro*, particularly upon TNF-α stimulation (data not shown). CCL5 has previously been described to multimerize with intact CXCL4 [34]. Therefore, binding assays were performed to corroborate a possible interaction. We found that both CXCL4^47–70^ and CXCL4L1^47–70^ directly and equally well interacted with CCL5 (Figure 10B). Considering previous reports of CCL5/CXCL4 multimers synergistically increasing monocyte attraction [34], we verified whether the interaction between the peptides and CCL5 could contribute to an enhanced monocyte influx into the tumors. It was demonstrated that adding CXCL4^47–70^ or CXCL4L1^47–70^ to CCL5 in the lower compartment of multiscreen chemotaxis chambers enhanced transendothelial migration of monocytic cells. Similar to the intact chemokines, CXCL4^47–70^ more efficiently stimulated monocytic cell chemotaxis compared to CXCL4L1^47–70^ (Figure 10C). These findings could explain the observed changes in the tumoral leukocyte population following peptide treatment.

## DISCUSSION

The chemokines CXCL4 and CXCL4L1 have both been attributed anti-tumoral activity as a result of angiostatic action, blockade of lymphatic vessel formation and the attraction of anti-tumoral lymphocytes [21, 23, 35, 36]. CXCL4L1 is considered to be a more powerful angiostatic and anti-tumoral agent than its homologue CXCL4, despite their striking 96% sequence identity [15, 35]. This functional difference has been linked to a single amino acid replacement, causing CXCL4L1 to adapt a unique carboxy-terminal 3D-structure with decreased affinity for GAGs [13]. However, the causal link remains puzzling, considering that CXCL4's capacity to bind GAGs, via a carboxy-terminal motif, has actually long been thought to be the main contributor to its angiostatic potential [18]. Moreover, carboxy-terminal peptides restricted to the last 24 amino acids of either CXCL4 (CXCL4^47–70^) or CXCL4L1 (CXCL4L1^47–70^) have been described to retain their anti-angiogenic and anti-tumoral properties [18, 19]. In an attempt to understand the working mechanism of both chemokines and thereby their future possible therapeutic applicability, we studied the angiostatic and anti-tumoral potential of these peptides more in depth. Interestingly, in the here studied MDA-MB-231 breast cancer model, CXCL4L1^47–70^ loses its relative potency over CXCL4^47–70^. This is in contrast with a previous study by our group demonstrating beneficial effects of CXCL4L1^47–70^, but not of CXCL4^47–70^, in the treatment of B16 melanoma. The anti-tumoral activity of CXCL4L1^47–70^ was ascribed to inhibition of angiogenesis and induction of apoptosis within the B16 melanoma tumor tissue [19]. In the MDA-MB-231 model, the anti-tumoral activity of the peptides probably depends more on the establishment of an anti-tumoral immune response and direct inhibition of tumor cell proliferation.

To reveal the molecular mechanisms involved we explored different possibilities, the first being direct molecular interaction between CXCL4^47–70^ or CXCL4L1^47–70^ and other cytokines (e.g. CCL5 and EGF). This heteromultimerization could change the receptor-activating properties of the cytokines. Second, binding to GAGs was assessed, which might affect kinetics of *in vivo* availability. GAG binding could also cause displacement of other cytokines from GAGs (decreasing cytokine receptor activation) or, on the contrary, could improve GAG interaction (enhancing cytokine activity). Finally, in addition to receptor-independent activities and GAG binding, a cellular receptor might be involved.

Concerning direct cytokine ligand interactions, both positive and negative interactions have been described for intact CXCL4 [33, 34, 37, 38] and its carboxy-terminal peptide [39]. CXCL4 has been demonstrated to counteract angiogenic VEGF and FGF2, by direct molecular interaction (heterodimerization) or GAG-displacement [37, 38]. In contrast, intact CXCL4 enhances monocyte chemotactic activity of CCL5 through heteromultimerization [34]. In this study we chose two cytokines important in our breast cancer model and verified interaction with CXCL4^47–70^ and CXCL4L1^47–70^: EGF as growth factor for the tumor cells and CCL5 as attractant of monocytes/macrophages. Both peptides bound with similar efficiency to immobilized EGF or CCL5. Furthermore, addition of CXCL4^47–70^ or CXCL4L1^47–70^ to CCL5 in a monocyte transendothelial migration assay increased monocyte chemotactic activity. On the other hand, we could not demonstrate binding or activation of CXCR3 by CXCL4^47–70^ or CXCL4L1^47–70^ suggesting that either no cellular receptor or a still unidentified receptor is activated. Alternatively, because the 47–70 carboxy-terminal domain contains the main GAG interaction site of CXCL4, we assessed heparin affinity of both peptides. We confirmed already published findings that CXCL4^47–70^ still interacts with heparin [18]. The heparin affinity of CXCL4^47–70^ was higher than that of CXCL4L1^47–70^. We therefore expect CXCL4^47–70^ to be retained longer at the site of injection and to exert its anti-tumoral activities over a longer time period. Our findings could explain the apparent difference between almost equal potency of the peptides *in vitro* (Figures 1–4) compared to more outspoken anti-tumoral activity for CXCL4^47–70^ in this *in vivo* breast cancer model.

Perception of the role of the immune system in tumor development has changed over time. Tumor inflammation is indeed the seventh hallmark of malignant disease, but the sizeable myeloid population present has been mostly observed to take on a tumor-promoting role rather than exert its protective, phagocytic and cytotoxic function [40–42]. This shift could be attributed to the phagocytes' versatility and their ability to adopt distinct phenotypes influenced by locally produced factors [43]. Rather than depleting the tumoral myeloid cell population, it could be beneficial to tackle its polarization and to have its pro-inflammatory actions complement anti-tumoral treatment. Therapeutic reversion of the tumor-associated leukocyte polarization could alter their impact on tumor progression, favoring anti-tumor immunity. Previously, CXCL4 has been reported to promote monocyte survival and macrophage activation [44]. This fits with the observed upregulation of macrophage markers in CXCL4^47–70^-treated tumors. The carboxy-terminal CXCL4 peptide could potentially maintain its effect on monocyte survival and activation, thereby leading to an expanding intratumoral macrophage population. As a single stimulus, CXCL4^47–70^ has been stated not to stimulate monocytic THP-1 migration [19]. Interestingly however, as already discussed, we revealed CXCL4^47–70^ and CXCL4L1^47–70^ to be able to multimerize with the monocyte chemoattractant CCL5, consequently enhancing migration of monocytic cells *in vitro*. Especially because the *in vivo* angiostatic effects were less prominent in this study, our results suggest that the CXCL4^47–70^-specific immunological effect might be a driving force behind its *in vivo* induced tumor growth retardation.

We did also confirm the conservation of angiostatic activity for both carboxy-terminal platelet factor peptides *in vitro* [17, 19]. Where our previous study has focused on the inhibitory effect of the peptides on endothelial cell migration, we now report anti-proliferative effects of CXCL4^47–70^ and CXCL4L1^47–70^ on both blood and lymphatic endothelial cells. Broadening the spectrum of activity to include blockade of lymphangiogenesis offers an interesting new therapeutic perspective as the lymphatic vasculature has been implied to be an escape route for metastatic tumor cells [21].

To date, anti-angiogenic therapy has not delivered on expectations in cancer research. Where anti-angiogenesis falls short, the immune system seems to play a critical role. Alternative avenues need to be explored. Here, we illustrate the anti-proliferative, angiostatic, inflammatory and overall anti-tumoral actions of carboxy-terminal peptides CXCL4^47–70^ and CXCL4L1^47–70^, which are in line with the effects of the original full-length mature proteins. However, the sum of their actions and eventual impact *in vivo* appear to be tumor model-dependent. Such a tumor-dependent therapy responsiveness to treatment would have important implications towards future therapeutic use of CXCL4, CXCL4L1 and derived peptides. In conclusion, this study illustrates, angiostatic chemokines such as CXCL4, CXCL4L1 and their derived peptides could fit the profile of multifunctional new anti-cancer therapeutics perfectly.

## METHODS

### Synthesis and purification of CXCL4^47–70^ and CXCL4L1^47–70^

The human carboxy-terminally derived peptides of the chemokines CXCL4 and CXCL4L1, namely CXCL4^47–70^ and CXCL4L1^47–70^, were synthesized by fluorenyl methoxycarbonyl (Fmoc) chemistry on a P11 peptide synthesizer (Activotec, Cambridge, UK) and purified by reversed phase chromatography as previously described [19]. Side-specific coupling of biotin to the N-terminus of the peptides was performed on the peptide synthesizer by activating and coupling biotin-ONp (Biotin p-nitrophenyl ester, Novabiochem, Darmstadt, Germany) on the Fmoc-deprotected N-terminus with the same program that was used for coupling the individual Fmoc-amino acids. This results in exactly one biotin group attached to the N-terminus of the peptide without modification of the side chains of lysine residues. Peptide purity, concentration and molecular mass were verified by ion trap mass spectrometry (Bruker, Bremen, Germany).

### Cell cultures

Preliminary tests were performed on fetal bovine aortic endothelial GM7373 cells [45], obtained from the N.I.G.M.S. Human Genetic Mutant Cell Repository (Institute for Medical Research, Camden, NJ) and grown in Eagle's MEM containing 10% fetal calf serum (FCS). HMVEC (Cell Systems, Kirkland, WA) and HLEC (Lonza, Verviers, Belgium) were grown in endothelial basal medium-2 (EBM-2) enriched with endothelial growth medium-2 MV Bulletkit (Lonza), following the manufacturer's instructions. In order to investigate the anti-tumoral effects of the chemokine-derived peptides, the EGF-dependent tumor cell line MDA-MB-231 was purchased from ATCC (Manassas, VA) and cultured in DMEM (Lonza) with 10% FCS. Human acute monocytic leukemia cells (THP-1) were cultured as previously described [46].

### Cell proliferation assays

#### MTT proliferation assay

First, HMVEC and HLEC were seeded at 5 × 10^3^ cells/well in EBM-2 + 1% FCS in a 96-well plate, whereas MDA-MB-231 were starved of growth factors overnight by replacing their growth medium with DMEM (serum-free) prior to seeding the tumor cells at 15 × 10^2^ cells/well in DMEM + 1% FCS. As for the rest of the assay, the same protocol applies to all cells. Once adherent, cells were incubated with different concentrations of the peptides CXCL4^47–70^ and CXCL4L1^47–70^ as single stimuli or in combination with recombinant human EGF (3 ng/ml; R&D Systems, Minneapolis, MN) at 37°C/5% CO_2_. All conditions were tested in triplicate or quadruplicate. After 3 to 4 days, stimulation medium was removed and a sterile MTT solution was added [0.04 g/100 ml thiazolyl blue (Sigma-Aldrich, St. Louis, MO) in 20% PBS/80% RPMI without phenol red (Lonza)] for 3 to 4 hours. The formed formasan crystals were dissolved by replacing the MTT solution with acidic propanol (0.04 M HCl, 0.1% (v/v) Nondet P-40 in absolute isopropanol) and shaking the plate, shielded from light, for 10 minutes. Optical density (OD) was determined at 570 nm and 630 nm. The obtained ODs were processed to proliferation indexes (PI), expressed relative to appropriate buffer controls, i.e. ΔOD570–630_X_ over ΔOD570–630_CO_.

#### GM7373 proliferation assay

Cell proliferation assays on GM7373 cells were performed as described [47]. Briefly, subconfluent cultures of GM7373 cells were seeded in 96-well dishes at 65 × 10^3^ cells/cm^2^. After 16 h, cells were incubated in triplicate in fresh medium containing 0.4% FCS plus FGF1 (30 ng/ml), FGF2 (10 ng/ml), FGF8 (30 ng/ml), EGF (10 ng/ml), VEGF (30 ng/ml), 12-O-tetradecanoyl phorbol 13-acetate (TPA, 10 ng/ml) or 10% FCS in the absence or in the presence of 400 ng/ml of CXCL4^47–70^ or CXCL4L1^47–70^. After 24 h, cells were trypsinized and counted in a Bürker chamber. Data were expressed as percentages of cell proliferation measured in the presence of the mitogenic stimulus alone.

### Transendothelial multiscreen chemotaxis assay

Monocyte chemotaxis assays were performed on 5 μm hydrophilic polycarbonate multiscreen-MIC plates (Millipore) using the human acute monocytic leukemia cells (THP-1). To promote efficient synergy between CCL5 on one hand and CXCL4, CXCL4^47–70^ or CXCL4L1^47–70^ on the other hand, the upper compartment of these plates was coated with a HMVEC monolayer. HMVEC were seeded on a 0.1% gelatin layer 24 hours prior to the chemotaxis assay. The following day, dilutions of recombinant human CCL5 (PeproTech, Rocky Hill, CT), natural human CXCL4 (isolated from stimulated platelets as previously described) [15], recombinant human CXCL4L1 (isolated from baculovirus-infected Sf9 cells as previously described) [23], synthetic CXCL4^47–70^, synthetic CXCL4L1^47–70^ (both produced as described above), or combinations thereof were added to the lower compartment of the plate, in a volume of 150 μl. After removal of the HMVEC medium, 0.35 × 10^6^ THP-1 cells were added to the upper wells, resuspended in phenol red-free RPMI + 0.1 g/100 ml BSA (endotoxin-free BSA; Sigma CatN° A8806) after centrifugation of the cells at 200g for 10 min. Plates were incubated at 37°C for 3 to 5 hours before quantifying the amount of cells that migrated towards the chemokine concentrations. For that purpose, we used the ATPlite kit (PerkinElmer, Waltham, MA) allowing quantitative luminescent detection of ATP, which is representative of THP-1 cell counts in the lower wells. This reaction was performed in a view white 96-well plate (PerkinElmer). A chemotactic index (CI) was calculated, defined as the ratio of luminescence after chemokine treatment and luminescence after control treatment.

### Intracellular p21 signaling assay

Signaling assays [23] were slightly adapted to the signaling molecules of interest in this study. HMVEC were stimulated with EGF (20 ng/ml) as a single stimulus or in combination with the peptides CXCL4^47–70^ and CXCL4L1^47–70^ (0.3 to 10 μg/ml) in order to evaluate their impact on the cell cycle-arresting p21. After 15 hours, the cells were washed three times with ice-cold PBS to stop the stimulation and lysis buffer [1 mM EDTA, 0.5% (v/v) Triton-X, 10 mM NaF, 150 mM NaCl, 1 mM DTT in PBS; pH 7.2–7.4] supplemented with a protease inhibitor cocktail (Sigma) was added. Cell residue was removed from the collected lysates by centrifugation (4°C, 10 min, 1666*g*). Lastly, the intracellular p21 content was determined by DuoSet® IC ELISA (cat n° DYC1047, R&D Systems) while, in parallel, total protein content was measured in a bicinchoninic acid (BCA) protein assay (Pierce, Rockford, IL).

### *In vivo* tumor experiment

SCID mice were bred in specific-pathogen-free conditions in the Experimental Animal Breeding Facility of the University of Leuven (KU Leuven) and fed sterilized food and water. All animal experiments were conducted in agreement with the Ethical Committee for Animal Care and Use of the KU Leuven and executed under license number LA1210243. 7- to 10-week-old SCID mice were used throughout the experiments. MDA-MB-231 cells in log growth phase (6 × 10^6^ cells resuspended in 200 μl of PBS) were injected subcutaneously (s.c.) on day 0 in the right dorsal flank. Animals were treated by intratumoral injection with control, 2.5 μg synthetic human CXCL4^47–70^ peptide or synthetic human CXCL4L1^47–70^ peptide three times a week starting from day 3. The external tumor volume was measured with calipers thrice a week and calculated using the formula (4πab2)/3 in which a and b are the largest and smallest radius, respectively. On day 27 or 28, mice were sacrificed and tumors were resected. The exact tumor volume of the resected tumor, based on three measured radii (a, b and c), was calculated by the formula (4πabc)/3. A portion of the tumors was stored at −70°C for generation of mRNA extracts and another part was processed for flow cytometry.

### Flow cytometric analysis of F4/80^+^ cells

For flow cytometry, single cell suspensions were prepared from tumor biopsies using a MACS dissociator (Myltenyi Biotec, Bergisch Gladbach, Germany) and a mixture of collagenase A and dispase. Remaining cell clumps were removed by passing the cell suspension through a nylon cell strainer (70 μm mesh). Remaining red blood cells were lysed in ACK lysis buffer (0.15 M NH_4_Cl, 0.1 mM Na_2_EDTA, 10 mM KHCO_3_; pH = 7.2). Subsequently monocytes were identified by flow cytometry using specific antibodies, namely rat anti-mouse F4/80-PE antibody (eBiosciences, San Diego, CA).

### Gene expression studies

The RNeasy Mini Kit (QIAGEN, Venlo, the Netherlands) was used for tumor mRNA extraction. The extracted mRNA was then converted to cDNA making use of the High Capacity cDNA Reverse Transcription Kit (Applied Biosystems, Foster City, CA). Quantitative polymerase chain reactions (qPCR) based on the TaqMan principle were conducted to compare gene expression between samples. Primers and probes (Table 2; Integrated DNA Technologies, Haasrode, Belgium), were diluted to the prescribed working concentrations in RNase-free water and added to the reaction mix in a MicroAmp® fast optical 96-well reaction plate (Applied Biosystems), together with 50 ng sample cDNA per reaction. The actual thermocycler program consisted of 2 minutes on 50 °C, 10 minutes on 95 °C and 40 cycles of 15 seconds on 95 °C followed by 1 minute on 60 °C. Analysis of the obtained C_T_ was based on the 2^−ΔΔ*C*^T^^ method, opting for 18S ribosomal 1 RNA as an internal control [48].

**Table 2:** Primer and probe sequences for qPCR assays

Gene	Primers (5′→ 3′)	Probe (5′→ 3′)
F4/80	GGAAGTGGATGGCATAGATGA ATTCACTGTCTGCTCAACCG	/HEX/AGTCTGGGA/ZEN/ ATGGGAGCTAAGGTCA/3IABkFQ/
IFN-γ	TCCACATCTATGCCACTTGAG CTGAGACAATGAACGCTACACA	/HEX/TCTTGGCTT/ZEN/ TGCAGCTCTTCCTCA/3IABkFQ/
TGM2	GGTTCATATCCAAGAGCATCAGG GTCAAGTTCATCAAGAGTGTGC	/FAM/TTCCATCCT/ZEN/ CGAACTGCCCAAAGT/3IABkFQ/
CD204	CACAAGGAGGTAGAGAGCAATG GCACGTTCAATGACAGCATC	/FAM/TCACAGCAC/ZEN/ TAAAAATGGCCCCTCC/3IABkFQ/
IL-12	TCCAGTCCACCTCTACAACA TGTCCTCAGAAGCTAACCATC	/FAM/ACGTCTTTC/ZEN/ TCCAGCTCCCACATG/3IABkFQ/
NOS2	CACTTCTGCTCCAAATCCAAC GACTGAGCTGTTAGAGACACTT	/HEX/TGAACAAGA/ZEN/ CCCAAGCGTGAGGAG/3IABkFQ/
CD11c	CTACCCGAGCCATCAATCAG GCTCTGCTTTCTACTGAGTTCA	/HEX/AGCCAGAAC/ZEN/ TTCCCAACTGCACA/3IABkFQ/
CCL5 (mu)	CCTCTATCCTAGCTCATCTCCA GCTCCAATCTTGCAGTCGT	/HEX/TCTTCTCTG/ZEN/ GGTTGGCACACACTT/3IABkFQ/
CCL5* (hu)	TGCCACTGGTGTAGAAATACTC GCTGTCATCCTCATTGCTACT	/FAM/ATCTGCCTC/ZEN/ CCCATATTCCTCGGA/3IABkFQ/
18S	ATCGCTCCACCAACTAAGAAC ACGGACAGGATTGACAGATTG	/FAM/ACCACCCAC/ZEN/ GGAATCGAGAAAGAG/3IABkFQ/

All primer/probe sets were supplied as PrimeTime qPCR assays by Integrated DNA Technologies (IDT)

* All assays were designed for murine (mu) gene products with the exception of this assay, designed to detect the human (hu) CCL5 cDNA.

### SPR equipment and reagents

SPR measurements were performed on a BIAcore X100 instrument (GE-Healthcare, Milwaukee, WI). Research-grade SA sensorchip was used. For the study of the interaction of heparin with CXCL4^47–70^ and CXCL4L1^47–70^, heparin was immobilized to a BIAcore sensorchip. Briefly, 13600 Da unfractionated heparin was biotinylated at its reducing end and immobilized onto the SA streptavidin-coated sensorchip. These experimental conditions allowed the immobilization of 200 resonance units (RU) equal to 14.7 fmol/mm^2^ of heparin. A sensorchip coated with streptavidin alone was used as a reference and for blank subtraction. The peptides were dissolved in 10 mM HEPES buffer pH 7.4 containing 150 mM NaCl, 3 mM EDTA, 0.05% surfactant P20 (HBS-EP+) and injected at different concentrations over the heparin or streptavidin surfaces for 2 min at a flow rate of 30 μl/min and then washed until dissociation was observed. After each run, the sensorchip was regenerated by injection of HBS-EP+ containing 2.0 M NaCl.

### GAG binding and heterodimerization assays in 96-well plates

GAG, EGF and CCL5 binding were quantified in 96-well plates following a similar protocol. GAG binding was evaluated by immobilizing low molecular weight heparin (Iduron, Manchester, UK) on EpranEx plates (BD Biosciences), whereas EGF as well as CCL5 recombinant human protein (R&D Systems and PeproTech, respectively) was immobilized on high protein-binding ELISA plates (Corning life sciences, Tewksbury, MA). In brief, 25 μg/ml GAG [diluted in assay buffer 100 mM NaCl, 50 mM NaAc, pH 7.2, and 0.2% (v/v) Tween-20], 2 μg/ml EGF or 50 ng/ml CCL5 (both diluted in PBS), was coated overnight at room temperature on 96-well plates. Plates were washed three times with either assay buffer or 0.5% (v/v) Tween 20 in PBS and blocked at 37°C with buffer enriched with either 0.2% (w/v) gelatin (heparin binding assay) or 0.1% (w/v) casein (EGF or CCL5 binding assay). Subsequently, a dilution curve of biotinylated CXCL4L1^47–70^ and CXCL4^47–70^ peptide was pipetted on the plate and incubated at 37 °C to allow interaction with heparin, EGF or CCL5. After washing, retained biotinylated peptide was detected by peroxidase-conjugated streptavidin. Peroxidase activity was quantified by measuring the conversion of 3,3′,5,5′-tetramethylbenzidine (Sigma-Aldrich) at 450 nm.

### Statistical analysis

The Mann-Whitney test was used as a non-parametric test to compare data from two independent groups, performed with the use of Statistica 12. When comparing three or more independent groups, a Kruskal-Wallis test was performed firstly to allow subsequent pairwise comparison. Linear regression was performed and evaluated using GraphPad Prism 5 software. Correlations were confirmed by calculation of the Spearman r coefficient with Statistica 12. Given data represent an average ± standard error of the mean (s.e.m.), unless stated otherwise. A *p*-value of less than 0.05 was considered to indicate a statistically significant difference. The number of independent experiments was indicated by n.

## SUPPLEMENTARY FIGURE


