# Supplementary material for: Angiostatic, tumor inflammatory and anti-tumor effects of CXCL447–70 and CXCL4L147–70 in an EGF-dependent breast cancer model
Source: Oncotarget. 2014 Oct 21;5(21):10916–33. doi: 10.18632/oncotarget.2538 (PMC4279419; doi:10.18632/oncotarget.2538)
Supplement: Supplementary file 1 [file oncotarget-05-10916-s001.pdf]

SUPPLEMENTARY FIGURE

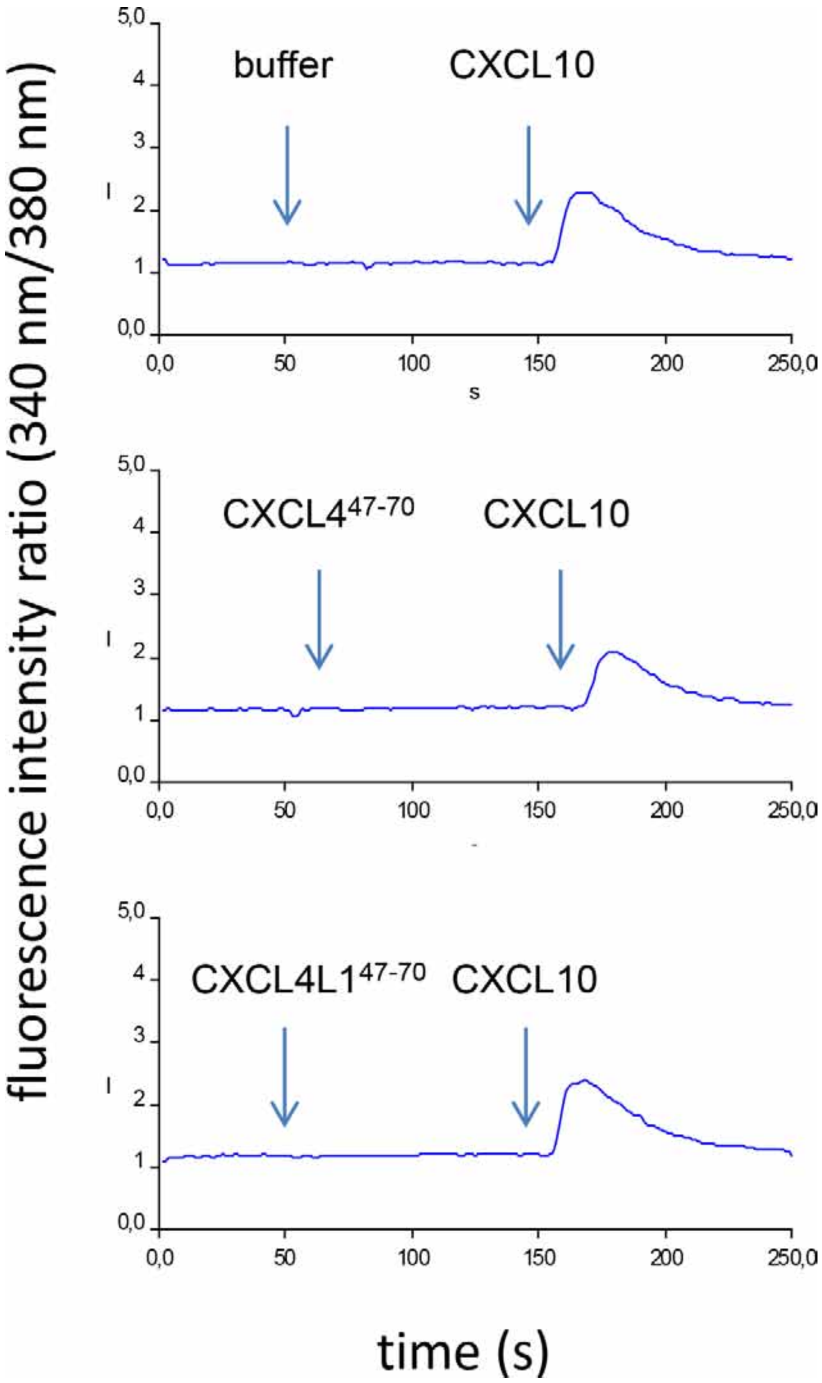

Supplemental Figure S1: CXCL4$^{47–70}$ and CXCL4L1$^{47–70}$ do not induce calcium signaling via CXCR3A and do not inhibit signaling by intact CXCL10. To study interaction of CXCL4$^{47–70}$ and CXCL4L1$^{47–70}$ with CXCR3A, CHO cells stably transfected with CXCR3A were loaded with the fluorescent dye fura 2 (Struyf et al. J. Immunol. 2009, 182:666). Changes in the intracellular Ca^{2+} concentrations ([Ca^{2+}]i) in response to CXCL4$^{47–70}$ or CXCL4L1$^{47–70}$ treatment were monitored using a spectrofluorometer. The line in the spectra indicates the fluorescence intensity ratio (i.e. the fluorescence intensity measured after excitation at 340 nm divided by the fluorescence intensity measured after excitation at 380 nm). The results shown (one representative experiment out of three) illustrate a desensitization experiment, in which CHO/CXCR3A cells were stimulated first with a peptide (2 µg/ml) and 100 s later with intact CXCL10 at a concentration (5 ng/ml) that induced a significant increase in [Ca^{2+}]i after prestimulation with buffer (upper panel). Neither of the two peptides induced a rise in [Ca^{2+}]i. When the cells were stimulated with 5 ng/ml intact CXCL10 100 s after peptide treatment, the response to intact CXCL10 was equal to the signal obtained after buffer stimulation. These results suggest that CXCL4$^{47–70}$ and CXCL4L1$^{47–70}$ do not interact with CXCR3A. Arrows indicate the time of addition of the stimulus.
